# Short-term treatment with eicosapentaenoic acid improves inflammation and affects colonic differentiation markers and microbiota in patients with ulcerative colitis

**DOI:** 10.1038/s41598-017-07992-1

**Published:** 2017-08-07

**Authors:** Anna Prossomariti, Eleonora Scaioli, Giulia Piazzi, Chiara Fazio, Matteo Bellanova, Elena Biagi, Marco Candela, Patrizia Brigidi, Clarissa Consolandi, Tiziana Balbi, Pasquale Chieco, Alessandra Munarini, Milena Pariali, Manuela Minguzzi, Franco Bazzoli, Andrea Belluzzi, Luigi Ricciardiello

**Affiliations:** 1 Department of Medical and Surgical Sciences, University of Bologna, Bologna, Italy; 2 Center for Applied Biomedical Research (CRBA), S.Orsola-Malpighi Hospital, University of Bologna, Bologna, Italy; 3 Department of Pharmacy and Biotechnology, University of Bologna, Bologna, Italy; 4 Institute of Biomedical Technologies–National Research Council (ITB-CNR), Segrate, Milan Italy; 5 Pathology Unit, S. Orsola-Malpighi Hospital, Bologna, Italy; 6 Gastroenterology Unit, S. Orsola-Malpighi Hospital, Bologna, Italy

## Abstract

Patients with long-standing ulcerative colitis (UC) have an increased colorectal cancer (CRC) risk. In this pilot study we evaluated the effect of Eicosapentaenoic acid as free fatty acid (EPA-FFA) supplementation on mucosal disease activity, colonic differentiation markers and microbiota composition in UC patients. Twenty long-standing UC patients in stable clinical remission and with fecal calprotectin (FC) > 150 µg/g were enrolled (T0) and supplemented with EPA-FFA 2 g/daily for 90 days (T3). Endoscopic and histologic disease activities were measured by Mayo and Geboes scores, respectively. HES1, KLF4, STAT3, IL-10 and SOCS3 levels were determined using western blotting and qRT-PCR, while phospho-STAT3 levels were assessed by western blotting. Goblet cells were stained by Alcian blue. Microbiota analyses were performed on both fecal and colonic samples. Nineteen patients completed the study; seventeen (89.5%) were compliant. EPA-FFA treatment reduced FC levels at T3. Patients with FC > 150 µg/g at T3 (n = 2) were assumed as non-responders. EPA-FFA improved endoscopic and histological inflammation and induced *IL-10, SOCS3*, HES1 and KLF4 in compliant and responder patients. Importantly, long-term UC-driven microbiota composition was partially redressed by EPA-FFA. In conclusion, EPA-FFA supplementation reduced mucosal inflammation, promoted goblet cells differentiation and modulated intestinal microbiota composition in long-standing UC patients.

## Introduction

Patients with ulcerative colitis (UC) have an increased risk to develop colitis-associated cancer (CAC) which is proportionally related to the duration and the extent of the disease^1^. Current strategies to prevent CAC development are mainly based on endoscopic surveillance in order to intercept and eradicate dysplasia which can evolve to a malignant transformation^2^. However, persistent active intestinal inflammation may hamper the identification of dysplastic areas during endoscopy. Thus, despite the reduction of advanced cancer incidence rates, obtained through a regular endoscopic surveillance, critical goals for CAC prevention remain to preserve a condition of histological remission^3, 4^, and to have predictive markers indicating those patients in whom endoscopic surveillance would be more effective. Fecal calprotectin (FC) is a cytosolic protein belonging to the S100 protein family, abundant in neutrophil granulocytes^5^, which represents a good predictor of endoscopic activity also in asymptomatic UC patients^6^.

Several relevant molecular mechanisms contribute to the malignant epithelial transformation during chronic intestinal inflammation. Among these, aberrant activation of the signal transducer and activator of transcription 3 (STAT3), Interleukin (IL)-10 deficiency or impaired function are critically involved in the onset of CAC^7, 8^.

Moreover, a thin and penetrable mucus layer, allowing a direct contact of bacteria with the epithelium, can lead to persistent colonic inflammation, thus promoting colon cancer development in UC patients^9^. Indeed, an over-growth of mucosal and fecal bacteria in inflamed colonic mucosa has been observed in UC patients, thus supporting a critical role of the intestinal microbiota in the pathogenesis of UC and progression to CAC^10, 11^.

The canonical *Notch* signalling pathway, through the modulation of the transcriptional target hairy and enhancer of split 1 (HES1), the antagonists atonal homolog 1 (HATH1) and kruppel-like factor 4 (KLF4) target, is crucial to preserve a proper intestinal differentiation^12, 13^. Our group recently proposed a tumor suppressor function of HES1 during CAC progression^14^, while the role of KLF4 in CAC is still controversial^15^.

The abnormal regulation of these transcriptional factors result in a compromised epithelial differentiation which can lead to an inefficient control of pathogenic microbes growth, favoring a tumor-prone microenvironment^16^.

The use of anti-inflammatory agents as tools for CAC prevention has been an intense focus of research^17, 18^. To date, there are no uncontested chemopreventive agents for CAC. We have recently demonstrated that a diet-containing highly-pure 1% eicosapentaenoic-acid as free fatty acid (EPA-FFA), an ω-3 polyunsaturated fatty acid (ω-3 PUFA), was able to prevent colon cancer initiation and promotion in the azoxymethan/dextran sodium sulfate (AOM-DSS) mouse model^14^. In the present exploratory study patients with long-standing UC in stable clinical remission and active inflammation identified by increased FC values, were supplemented with EPA-FFA in order to test its effects on relevant mechanisms associated with UC disease and progression to CAC.

## Results

### EPA-FFA supplementation induces FC reduction and favors endoscopic and histological remission

In this study, twenty patients with long-standing UC were enrolled. After baseline colonoscopy, one patient presented a clinical relapse before starting EPA-FFA supplementation, and was excluded from the trial. Nineteen patients completed the study. The clinico-pathological features of all patients (n = 19) at T0 are shown in Table 1. Noteworthy, during EPA-FFA supplementation, no clinical relapse was observed.

**Table 1:** Clinico-pathological characteristics of patients at baseline (T0; n = 19).

Patients' Characteristics	
Age, years median (range)	45 (23–80)
Male, n (%)	13 (68.4)
Current smokers, n (%)	1 (5.3)
BMI median (range)	24.16 (18.5–34)
Duration of UC, years median (range)	12 (8–27)
Time of remission, months median (range)	24 (4–60)
Fecal Calprotectin, (µg/g) median (range)	220 (150–300)
C-Reactive Protein, (mg/L) median (range)	0.3 (0.04–1.25)
SCCAI clinical score > 3 n (%)	0 (0)
Mayo endoscopic sub-score ≥ 1 n (%)	13 (68.4)
Geboes histological score ≥ 3.1 n (%)	7 (36.8)
**Concomitant medication, n (%)**	
Mesalamine	11 (57.9)
Azathioprine	2 (10.5)
Mesalamine + Azathioprine	4 (21.0)
Anti-TNFα	1 (5.3)
None	1 (5.3)

**Figure 1:**
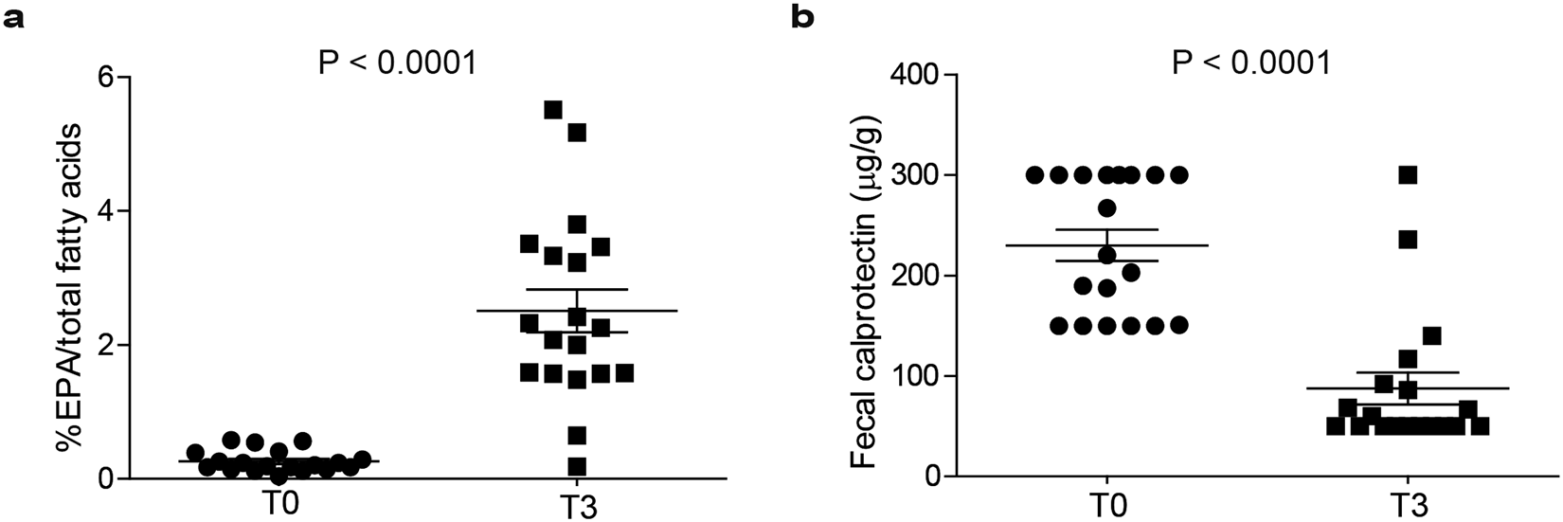
(**a**) Eicosapentaenoic acid (EPA; C20:5 n-3) percentage in RBCs and (**b**) FC levels (µg/g) (B) in all patients (n = 19) at T0 and T3. Statistical significance was calculated using the paired two-tailed t-test. Data are shown as mean ± SEM.

Fatty acids composition was evaluated on RBC-purified membranes. Compared to T0, EPA-FFA supplementation led to a significant increase of EPA (P < 0.0001; Fig. 1a). The mean percentage values of EPA content changed from 0.26 at T0 to 2.51 at T3. Capsules counting revealed that seventeen patients were adherent to treatment with an overall compliance of 89.5%. Since EPA can be converted into the ω-3 PUFA docosahexaenoic acid (DHA) *in vivo* through docosapentaenoic acid (DPA)^19^, we also measured the overall ω-3 PUFAs content including EPA, DPA and DHA, in our patients. Interestingly, the combined percentage content of EPA, DPA and DHA was significantly increased at T3 compared to T0 (P < 0.0001; Supplementary Figure S1a), while the percentage content of ω-6 PUFAs (arachidonic + linoleic acids) was unchanged upon EPA-FFA supplementation (Supplementary Figure S1b).

Importantly, a significant reduction of FC at T3 was observed (P < 0.0001; Fig. 1b). The mean FC values changed from 230 at T0 to 87.7 µg/g at T3. No side effects or serious adverse events were reported during the trial. Two patients maintained FC levels >150 µg/g at T3 after treatment and were considered non-responders. EPA-FFA treatment significantly promoted endoscopic and histological remission in compliant and responder patients (n = 15).

**Figure 2:**
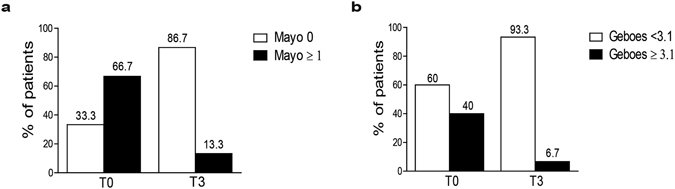
(**a**) Mayo endoscopic score and (**b**) Geboes histological score in compliant and responder patients (n = 15) at T0 and T3. Data are presented as percentage of patients according to Mayo and Geboes cut-offs.

Indeed, compared to baseline, endoscopic improvement was observed in 8 patients while no variations were observed in 7 (P = 0.004; Fig. 2a). Moreover, a resolution of histological inflammation at T3 was observed in 5 patients, while the histological score remained unchanged in 10 (P = 0.03; Fig. 2b). Endoscopic and histological worsening was not observed.

### EPA-FFA supplementation induces both *IL-10* and *SOCS3* expression reducing STAT3 activation

**Figure 3:**
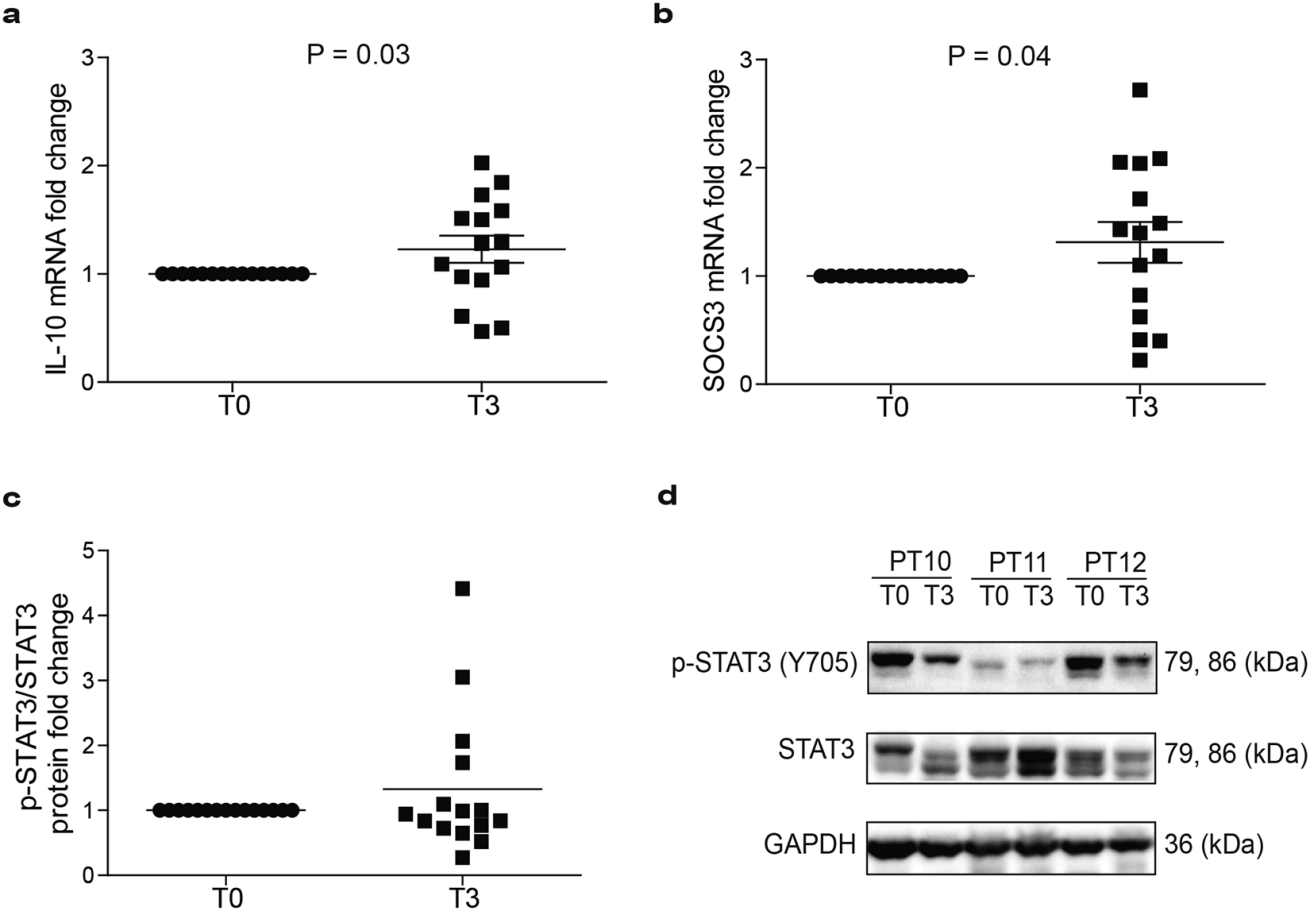
mRNA expression levels of **(a)** *IL-10* and **(b)** *SOCS3*. Protein levels of **(c)** p-STAT3/STAT3 on homogenized sigmoid colon tissues in compliant and responder patients (n = 15) at T0 and T3. Statistical significance was obtained using one-sample two-tailed t-test. Data are shown as mean of square root transformed values ± SEM. **(d)** Western blot representative images of p-STAT3 (Y705) and STAT3 at T0 and T3 (n = 3).

To elucidate the mechanisms responsible for the protective effect of EPA-FFA in patients with long-standing UC, we investigated the modulation of the IL-10/STAT3/SOCS3 axis in compliant and responder patients (n = 15). Compared to T0, we observed a concomitant significant up-regulation of *IL-10* (P = 0.03; Fig. 3a) and *SOCS3* (P = 0.04; Fig. 3b) mRNA levels at T3 associated with an increasing trend in *IL-22* mRNA (Supplementary Figure S2). Otherwise, no significant differences in IL-10 and SOCS3 protein expressions were observed (Supplementary Figure S3a and b). Since STAT3 represents one of the major regulators of *SOCS3*, we decided to characterize STAT3 activation in these patients. Treatment with EPA-FFA reduced STAT3 Tyr705 phosphorylation (p-STAT3) in 60% of patients (9/15) (Fig. 3c and d), while not affecting STAT3 transcription (Supplementary Figure S4). Noteworthy, 4/5 patients showing highest levels of p-STAT3 at T3 were poor compliant patients with lower percentage of EPA in RBC after supplementation. These data suggest that over-expression of *SOCS3* following EPA-FFA supplementation, probably as a downstream effect of *IL-10* induction, reduces STAT3 activation, in particular in patients with highest percentage of EPA in RBC membranes. Correlation analyses revealed a significant positive correlation at T3 between transcriptional levels of *SOCS3* and both *IL-10* mRNA levels (P = 0.02; Supplementary Figure S5a) and p-STAT3 protein (P = 0.03; Supplementary Figure S5b), thus supporting our hypothesis.

### EPA-FFA supplementation modulates HES1 and KLF4 and stimulates goblet cell differentiation

**Figure 4:**
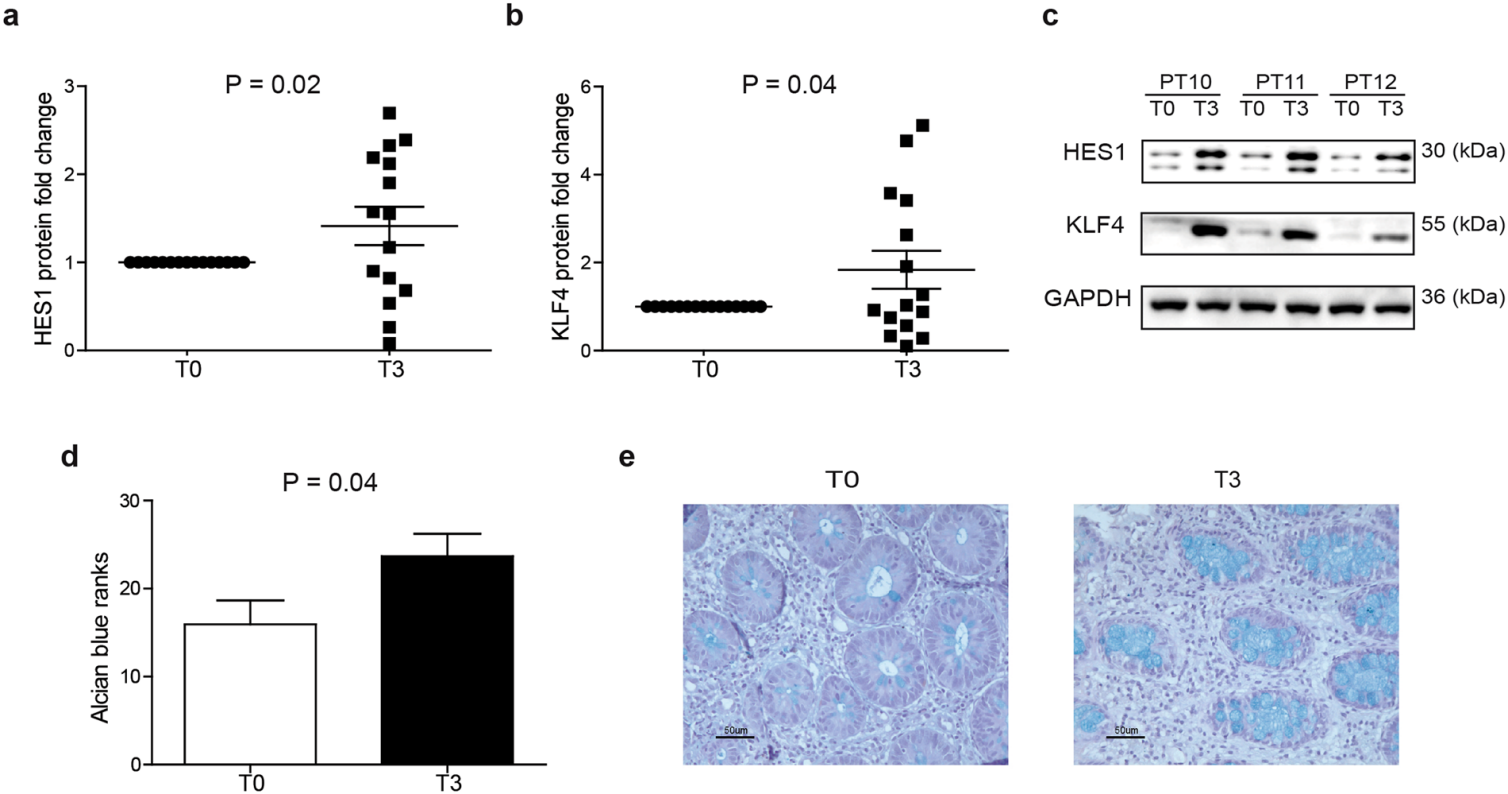
Protein expression levels of **(a)** HES1 and **(b)** KLF4 on homogenized sigmoid colon tissues in compliant and responder patients (n = 15) at T0 and T3. Statistical significance was measured using one-sample two-tailed t-test. Data are shown as mean of square root transformed values ± SEM. **(c)** Western blot representative images of HES1 and KLF4 at T0 and T3 (n = 3). **(d)** Alcian blue ranks and **(e)** representative images of goblet cells staining at T0 (left panel) and T3 (right panel). Statistical significance for Alcian blue ranks was calculated using the paired two-tailed t-test. Data are shown as mean of ranks ± SEM.

*Notch* signaling, through the modulation of the transcriptional targets HES1 and KLF4, is crucial to preserve a proper balance between the absorptive and the secretory cell lineages of the intestine^13, 20^. We observed a significant up-regulation of HES1 (P = 0.02; Fig. 4a and c) and KLF4 proteins (P = 0.04; Fig. 4b and c) in patients with long-standing UC at T3 compared to T0, while no differences were observed at mRNA level (Supplementary Figure S6a and b). Correlation analysis indicated that these two transcription factors positively correlate with each other (P = 0.0007; Supplementary Figure S7). Importantly, although no variations in the MUC2 mRNA (Supplementary Figure S8a) and protein were found (Supplementary Figure S8b), compared to T0, in which goblet cells depletion was found in 20% of patients, daily supplementation of EPA-FFA for 3 months was associated with a significant increased number of goblet cells in the colon (P = 0.04; Fig. 4d and e). Thus, our results unveil a role of EPA-FFA in improving secretory lineage differentiation and intestinal epithelial cells turnover through simultaneous induction of KLF4 and HES1. We found no differences in terms of intestinal proliferation measured by Ki-67 (Supplementary Figure S9a), *c-MYC* (Supplementary Figure S9b) and *LGR5* upon EPA-FFA supplementation (Supplementary Figure S9c).

### EPA-FFA modulates the gut microbiota composition in UC patients

Given the critical role of intestinal microbial imbalance in the pathogenesis of UC, the fecal and mucosal microbiota compositions were also assessed in our patients.

Sequences are available at the following MG-Rast link:

http://metagenomics.anl.gov/mgmain.html?mgpage=project&project=mgp80642.

**Figure 5:**
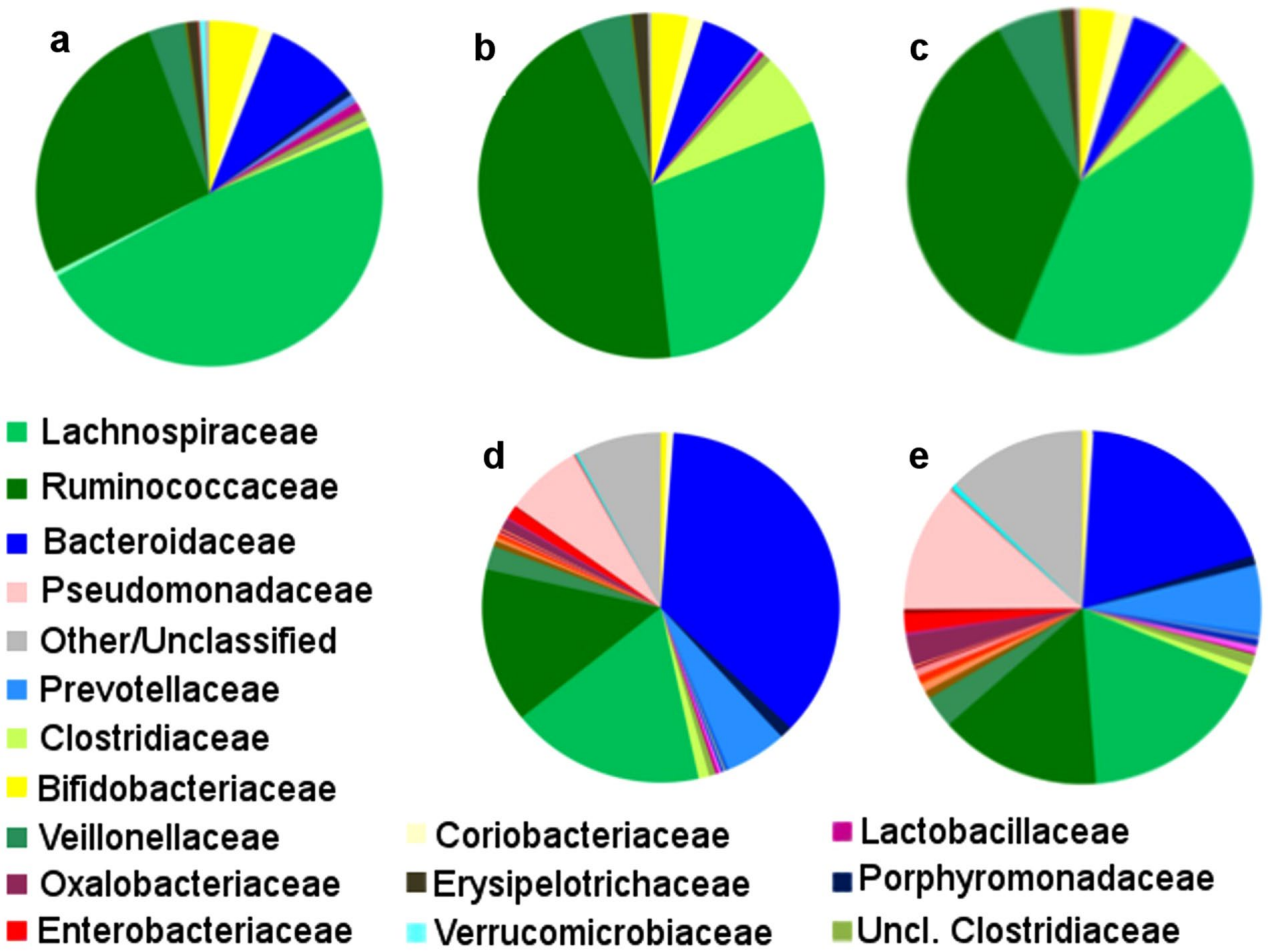
Median fecal microbiota composition at family level in **(a)** healthy adults, **(b)** UC patients at T0, **(c)** UC patient at T3 and colon biopsies of **(d)** UC patients at T0 and **(e)** UC patients at T3, represented as pie chart, in available samples from compliant and responder patients. Average relative abundance of families representing at least 0.2% of the total microbiota in at least 10% of the sequenced samples are showed. Color code for the most abundant bacterial families (present at an average abundance > 1% in at least one group of samples) is reported in approximate decreasing abundance order. Mann-Whitney U test was used to test differences among median groups.

To identify the main microbiota dysbioses associated with the long-term UC disease, the fecal microbiota composition of UC patients at T0 was compared to that of a group of Italian healthy adults (age 22–48 years, enrolled in the same geographical area of the UC patients)^21^. An enrichment of the families *Clostridiaceae* (4.7 *vs*. 1%, P = 0.003; in particular genus *SMB53*, P = 0.001) and *Ruminococcaceae* (35.7 *vs*. 24.1%, P = 0.008), and depletion of *Verrucomicrobiaceae* (0 *vs*. 0.4%, P = 0.002; in particular genus *Akkermansia*, 0 *vs*. 0.4%, P = 0.002), *Peptostreptococcaceae* (0 *vs*. 0.3%, P = 0.0009) and *Porphyromonadaceae* (0 *vs*. 0.5%, P = 0.006; in particular genus *Parabacteroides*, 0 *vs*. 0.5%, P = 0.006) families was found in UC patients at T0 (Fig. 5a and b). Noteworthy EPA-FFA supplementation increased *Porphyromonadaceae* (from 0 to 0.2%) and decreased *Ruminococcaceae* (from 35.7 to 28%) (Fig. 5b and c) in feces of UC patients. In addition, EPA-FFA had also effects on mucosal microbiota of UC patients by decreasing the abundance of mucosal-adherent members of the *Bacteroidaceae* family (in particular belonging to the genus *Bacteroides*, 27.4 *vs*. 14.7%) (Fig. 5d and e).

## Discussion

Different therapeutic approaches have been tested for CAC prevention in patients with Inflammatory bowel disease (IBD) over the years. Importantly, an increasing number of data obtained from *in vitro* experiments, as well as, animal and clinical studies support a protective role for ω-3 PUFAs (EPA and DHA) in gastrointestinal cancer prevention including CRC (as reviewed by Eltweri *et al*.^22^).

However, data on pharmacological and natural compounds as anticancer agents in IBD patients are elusive and still inconsistent^23^. It is well known that symptoms in IBD and serum biomarkers do not always properly mirror the inflammatory degree of the mucosa^24^. FC is becoming the most useful non-invasive tool for monitoring the inflammatory status of the mucosa and the response to therapy, as well as for predicting clinical relapse in IBD patients^25^.

In this pilot study we tested, for the first time, the effects of EPA-FFA on asymptomatic patients with long-standing UC in clinical remission who retained high FC levels (> 150 µg/g) despite stable maintenance therapy. We found that short-term EPA-FFA supplementation at a dosage of 2 g/daily was associated with a significant increase of EPA and overall ω-3 PUFAs content (EPA, DPA and DHA) into RBCs, suggesting that EPA was incorporated by most of patients (17/19) and efficiently converted into DPA and DHA.

Although the primary end-point of this study was not to test the clinical benefit of EPA-FFA but to explore its effect on mucosal inflammation and new potential chemopreventive mechanisms during long-standing UC, we found that short-term EPA-FFA supplementation reduced mucosal inflammation (with a significant drop in FC) favoring an improvement of both endoscopic and histological inflammation in almost all patients. Since FC levels have been demonstrated to correlate with the intensity of the neutrophilic infiltrate^12^, our results, supported by histological evaluation, indicate that EPA-FFA improved the inflammatory state in patients with long-standing UC. We believe that our results could be explained, at least in part, by the free-fatty acid-highly pure formulation of EPA used in this study.

Previous evidence support a protective role for ω-3 PUFAs intake including both EPA and DHA in the prevention of CRC in different settings^26–28^. However, data from ω-3 PUFAs supplementation in patients and murine models of UC are still controversial^29–31^, and the impact of dietary ω-3 PUFAs supplementation for CAC prevention is poorly defined.

Given the increased content of ω-3 PUFAs in our patients, it is reasonable to speculate that the observed protective effects may be due to both EPA and DHA. Noteworthy, we found no relevant differences in the ω-6 PUFAs content upon EPA-FFA supplementation. This result could be explained by the unchanged dietary habits of enrolled patients during the study. Strikingly, the increased ω-3 PUFAs content was sufficient to induce a relevant protective response in UC patients, while possibly maintaining the same ω-6 PUFAs content as previously suggested^32^.

In this study, in order to characterize the EPA-FFA short-term effects in long-standing UC patients, we first focused on the effects of EPA-FFA supplementation on IL-10/STAT3/SOCS3 signalling. The role of STAT3 during UC is actually controversial. Indeed, studies on animal models of IBD suggested both a deleterious and protective role of STAT3 hyperactivation during colitis^33, 34^. Importantly, increased levels of phospho-STAT3 were detected in patients with active UC, as well as in dysplasia and cancer, while a progressive decreasing trend of SOCS3 levels was observed from low-grade dysplasia to UC-CRC^35^. However, more recent evidence obtained in UC patients supported a role of SOCS3 over-expression in short-term disease relapse and mucosal inflammation impairing STAT3 activation^36, 37^. In this study, we found a concomitant significant up-regulation of *IL-10* and *SOCS3* mRNA upon EPA-FFA supplementation with a reduction of STAT3 activation in most of the patients with highest EPA percentage levels at T3. However, no changes in IL-10 and SOCS3 proteins were appreciated upon EPA-FFA supplementation in our patients. As previously suggested by literature data, we hypothesized that multiple post-transcriptional mechanisms may contribute to regulate SOCS3 and IL-10 proteins, thus explaining the absence of a correlation between changes in their mRNA and protein levels^38–42^.

Importantly, considering responders, we did not observe endoscopic or histological worsening in any patient. Thus, our data support a protective role of EPA-FFA during UC remission by turning off STAT3 activation through *SOCS3* transcriptional induction.

*Notch* signalling is also a key determinant for sustaining intestinal epithelial cells differentiation and turnover, for the integrity of the mucosal barrier, as well as for regulating malignant epithelial transformation in the colon^20^. Evidence show possible oncogenic and tumor suppressor activities of HES1 and KLF4 in sporadic settings, respectively^43, 44^. We previously showed in the AOM-DSS mouse model a loss of *Notch1* signalling during CAC development partially counteracted by EPA-FFA supporting a tumor-suppressor role of this pathway during inflammation-induced intestinal tumorigenesis^14^. Accordingly, Garg and colleagues previously demonstrated in the same animal model, that Matrix metalloproteinase-9 (MMP-9), activating *Notch1* signalling and controlling p53 cascade, exerts a strong protective effect toward CAC development^45^. Otherwise, in a recent *in vitro* work conducted by our research group, we observed a MMP-9-dependent activation of *Notch1* signalling in CRC cells exposed to a conditioned medium (CM) containing multiple pro-inflammatory cytokines secreted by activated macrophages. The activation of MMP-9/Notch signalling was associated with increased CRC cells invasiveness, suggesting a tumor-prone role of *Notch1* signalling in sporadic CRC. Interestingly, EPA-FFA pre-treatment of CM-exposed CRC cell lines led to reduced invasion through a *Notch1* signalling switch off^46^. These results, as recently reviewed by our research group^47^, clearly indicate that the cell response to *Notch* signalling activation is not univocal resulting in oncogenic or tumor-suppressive mechanisms depending on the specific pathological context.

In this study we demonstrated that EPA-FFA modulated intestinal differentiation inducing both HES1 and KLF4 proteins and increasing the number of goblet cells.

Patients with UC in remission are generally characterized by an intact mucus layer, although a defective and penetrable intestinal barrier could be retained in some cases^48^. KLF4 has a crucial role on both maturation and differentiation of goblet cells in the colon^49^, and a critical role for IL-10 in the regulation of goblet cells activation during inflammation has been also previously described^50^. Moreover, microbiota analysis performed in our study shows that the gut microbiota population constituents present in the UC group at T0 were partly modulated by the EPA-FFA treatment. Indeed, the *Porphyromonadaceae* genus *Parabacteroides*, known to be decreased in UC^51^, was significantly increased in T3 samples compared to T0. Also EPA-FFA showed the capability to reduce the fecal amount of *Clostridium spp*. compared to T0. Interestingly, these proteolytic microorganisms were known to induce mucolytic metabolism in other species, i.e. *Bacteroides* ^52^. Noteworthy, mucosal-adherent members of the *Bacteroides* genus, known to include mucolytic species, were found to be decreased after EPA-FFA treatment, possibly contributing to the protection of the epithelium. Thus, we hypothesize that the ability of EPA-FFA treatment to promote goblet cells population could be a result of multiple mechanisms including the induction of KLF-4 and IL-10, as well as the reduction of mucolytic bacteria. However, despite the impact of EPA-FFA supplementation on important modulatory functions in UC patients, our study has some limitations and should be taken with caution. Firstly, this explorative study involved a small number of subjects. Secondly, no patients received placebo and we exclusively tested a single dosage of EPA-FFA. Thirdly, we cannot completely rule out that the clinical outcome observed in our patients could be the result of a potential combined effect between EPA-FFA and stable maintenance of UC therapies, such as 5-aminosalicylic acid which is taken by most of the recruited patients.

In conclusion, in this pilot study EPA-FFA improved endoscopic and histological inflammation, affected the IL-10/STAT3/SOCS3 cascade, stimulated goblet cells differentiation and modulated the long-term UC-related colonic alterations of intestinal microbiota. Future larger placebo controlled trials should be conducted in order to confirm these results and to evaluate long-term effects of EPA-FFA supplementation on disease relapse and CAC risk.

## Methods

### Study design

Eligible patients were asymptomatic subjects aged 18–70 years with long-standing (≥ 8 years) UC, in stable clinical remission (simple clinical colitis activity index; SCCAI = 0), and FC levels higher than 150 µg/g^53^. Patients were included in the study after signing the informed written consent. Concomitant stable therapies for UC (mesalamine, immunomodulators and/or biological drugs) without modifications in the previous 3 months were allowed. Exclusion criteria were: (1) recent use of steroids (< 2 months) or other experimental drugs (< 3 months); (2) concomitant use of anticoagulants; (3) probiotic use; (4) pregnancy or breast-feeding; (5) known or suspected hypersensitivity to eicosapentaenoic acid or ω-3 PUFAs; and (6) severe co-morbidities. Subjects were given oral supplementation of 2 g/daily (two 500 mg capsules twice a day) of EPA-FFA (ALFA™, SLA Pharma AG, Switzerland) for 90 days. During the study, subjects were asked to keep their dietary habits. Patients underwent endoscopic examination at enrollment (T0) and after 90 days of EPA-FFA supplementation (T3). Six biopsies were taken from the sigmoid colon at each time point. Blood samples were obtained for isolation of peripheral erythrocytes. Adherence to EPA-FFA supplementation was evaluated both by capsule counting and assessing EPA incorporation into red blood cell (RBC) membranes. Compliant patients were considered those who consumed at least 80% of the capsules, without interruption of the protocol for more than 14 consecutive days. The study was conducted in accordance to the Declaration of Helsinki and approved by the Ethic Committee of the S.Orsola-Malpighi Hospital (Bologna, Italy).

The trial was registered on ClinicalTrials.gov with Identifier: NCT02069561 on 19/02/2014. (https://clinicaltrials.gov/ct2/show/NCT02069561)].

### Fecal calprotectin dosage

Fecal samples were collected within 24 hours before endoscopy and stored at 2–8 °C until assaying. Quantification of FC was carried out using CalFast (Eurospital, Trieste, Italy) according to the manufacturer’s protocol. FC values > 150 μg/g were considered predictive of mucosal endoscopic activity as previously demonstrated^53^.

### Endoscopic and histological evaluation

Two investigators (L.R., E.S.) performed all endoscopies. According to the Mayo endoscopic sub-score, a cut-off ≥ 1 was used to discriminate the presence of endoscopic inflammation^54^. Histological activity was assessed by one expert blinded pathologist (T.B.) and scored according to the Geboes grading system^55^. A Geboes cut-off score ≥ 3.1 was assumed to define active histological inflammation^56^. When biopsies showed different degrees of activity, the highest degree of inflammation was considered.

### Acidic mucins quantification

Formalin-fixed and paraffin-embedded (FFPE) biopsies were de-waxed in toluene for 10 minutes, rehydrated, placed in the Alcian blue solution (Alcian blue 8GX in 3% acetic acid solution pH 2.5) for 30 minutes and counterstained with hematoxylin. For analysis, slides were placed in order of increasing Alcian blue staining intensity using a rank order scoring system (1 = lower rank; 36 = higher rank). Rank ordering method has been shown to be better than categorical scoring system to identify subtle differences between groups^57^.

### Immunoistochemistry

Immunohistochemistry (IHC) was performed on FFPE colonic sections. Slides were dewaxed, subjected to endogenous peroxidase inhibition, rehydrated and treated with citrate buffer (pH 6.0) at 120 °C for 15 minutes for antigen retrieval. Then, slides were incubated overnight at +4 °C with the monoclonal antibodies against Ki-67 and MUC2 (Supplementary Table 1). After incubation with secondary antibody Rabbit/Mouse (1:1000, DAKO EnVision™ System Peroxidase), the signal was detected with diaminobenzidine (DAB) (Sigma-Aldrich, Saint Louis, Missouri, USA). Percentages of Ki67 positive nuclei and MUC2 positive DAB areas were quantified using ImageJ software (NIH, Bethesda, MD, USA).

### Membrane fatty acid analysis

Membrane fatty acids content was measured in RBCs. Lipids extraction from RBC membranes, phospholipids separation and sample preparation were performed as previously described^58^. Extracted fatty acid methyl-esters were then analyzed by gas-chromatography mass-spectrometry (GC-MS). Fatty acid levels were expressed as relative percentages of total fatty acids.

### Western Blotting

Total protein lysates were isolated from biopsies by sonication in RIPA buffer. Forty µg of proteins for each sample were separated on a 4–12% NuPAGE Novex Bis-Tris Gels (Invitrogen™, Thermo Fisher Scientific, Waltham, Massachusetts, USA) in MOPS buffer (Novex™, Thermo Fisher Scientific) and transferred onto nitrocellulose membrane. After blocking, membranes were incubated overnight at +4 °C with primary antibodies against HES1, KLF4, phosphorylated STAT3 (Y705), STAT3, IL-10, SOCS3 and GAPDH (Supplementary Table 1). After incubation with appropriate secondary Horse-Radish-Peroxidase (HRP) conjugated antibodies (GE Healthcare Life Sciences, Little Chalfont, United Kingdom), the signal was detected with a luminol enhancer solution (WESTAR EtaC, Cyanagen, Bologna, Italy) and images were acquired using the Chemidoc^TM^ XRS + (Biorad, Hercules, CA, USA). Densitometric analysis performed using Image Lab™ software.

### Gene expression analysis

Total RNA was extracted from biopsies using Trizol® (Ambion, Thermo Fisher Scientific). One µg of total RNA was converted to cDNA using the High-Capacity RNA-to-cDNA™ Kit (Applied Biosystems™, Thermo Fisher Scientific) according to the manufacturer’s instructions. qRT-PCR reactions were performed in duplicate on a MX3000p QPCR thermal cycler (Stratagene, San Diego, CA, USA) using the SYBR® Select Master Mix for CFX (Applied Biosystems™, Thermo Fisher Scientific) and the specific primers for *IL-10*, *IL-22*, *LGR5*, *C-MYC*, *MUC2*, *HES1* and *KLF4*. The primers sequences are listed on Supplementary Table 2. mRNA expressions of *SOCS3* and *STAT3* were analyzed using a 5′ nuclease probe (Assay ID: Hs.PT.58.4303529; Integrated DNA Technologies, Coralville, Iowa, USA) and the Taqman® gene expression assay (Hs00374280_m1; Thermo Fisher Scientific), respectively. Fold induction levels were obtained using the 2^−ΔΔCt^ method by normalizing against the reference gene *RPS9*.

### Microbiota analysis

Fecal samples were collected prior to the endoscopic preparation while mucosal samples were taken during endoscopy.

Total bacterial DNA was extracted from feces using QIAamp DNA Stool Mini Kit (QIAGEN, Hilden, Germany) and from biopsies using DNeasy Blood & Tissue Mini Kit (Qiagen). Due to a poor quality or quantity of extracted DNA, data on fecal and mucosal microbiota were available from 14 and 16 of the 19 patients included in the study, respectively. For all samples the V3–V4 region of the bacterial 16S rRNA gene was amplified and sequenced using the Illumina platform (Illumina, San Diego, CA) using a 2 × 300 bp paired-end protocol. Indexed libraries were pooled at equimolar concentrations, denatured and diluted to 6 pmol/L before loading onto the MiSeq flow cell. Raw sequences were processed using a pipeline combining PANDAseq [S6] and QIIME [S7]. High-quality reads were binned into operational taxonomic units (OTUs) at a 0.97 similarity threshold using UCLUST [S8] and a “de novo” approach. Taxonomy was assigned using the RDP classifier against the Greengenes database (May 2013 release). All singleton OTUs were removed in an attempt to discard the majority of chimera sequences. Relative abundance profiles at family or genus level were obtained and plotted. For fecal microbiota analysis, a comparison with a control population of Italian healthy adults enrolled in a previous study was also performed^21^. Fecal samples from healthy subjects were collected and processed using the same procedures applied for UC patients recruited in this study.

### Statistical analysis

Data were analyzed with Graphpad 5.0 Software (GraphPad Software Inc., CA, USA) and Statistix 9.0. The means of two matched groups (T0 *vs*. T3) were compared using the paired two-tailed t-test. For statistical analysis (based on fold-changes) the mean of T0 samples was assumed as 1 and two-tailed one-sample t-test was used to compare differences between T0 and T3. Sign test, a test for analyzing simple +/− differences between paired comparisons^59^, was used to analyze differences in the Mayo sub-score and Geboes score. Correlation analyses were carried out using Spearman’s correlation coefficient (r_s_). For qRT-PCR and western blot analyses data were presented upon square-root transformation. For microbiota analysis, median differences among groups were tested using a non parametric approach (Mann-Whitney U test); P values were corrected for multiple comparisons using the Benjamini-Hochberg method. P values < 0.05 were considered statistically significant.

All data generated or analysed during this study are included in this published article (and its Supplementary Information files).

## Electronic supplementary material

Supplementary information
