# Supplementary material for: Short-term treatment with eicosapentaenoic acid improves inflammation and affects colonic differentiation markers and microbiota in patients with ulcerative colitis
Source: Sci Rep. 2017 Aug 7;7:7458. doi: 10.1038/s41598-017-07992-1 (PMC5547132; doi:10.1038/s41598-017-07992-1)
Supplement: Supplementary file 1 — Supplementary information [file 41598_2017_7992_MOESM1_ESM.pdf]

Supplementary Tables and Figures

Short-term treatment with eicosapentaenoic acid improves inflammation and affects colonic differentiation markers and microbiota in patients with ulcerative colitis

Anna Prossomariti[1,2#], Eleonora Scaioli[1#], Giulia Piazzi[2], Chiara Fazio[1,2], Matteo Bellanova[1], Elena Biagi[3], Marco Candela[3], Patrizia Brigidi[3], Clarissa Consolandi[4], Tiziana Balbi[5], Pasquale Chieco[2], Alessandra Munarini[1,2], Milena Pariali[2], Manuela Minguzzi[1,2], Franco Bazzoli[1], Andrea Belluzzi[6], Luigi Ricciardiello[1]*

Table S1. List of primary antibodies used for IHC and western blot analyses

Primary antibodies	Supplier	Catalog number	Species	Type	Dilution
**Ki-67**	Dako	M7240	Mouse	Monoclonal	1:100
**MUC2**	Abcam	ab134119	Rabbit	Monoclonal	1:500
**HES1**	Pierce™	PA5-28802	Rabbit	Polyclonal	1:3,000
**KLF4**	Pierce™	PA5-35303	Rabbit	Polyclonal	1:1,000
**p-STAT3 (Y705)**	Cell Signaling	#9145	Rabbit	Monoclonal	1:1,000
**STAT3**	Cell Signaling	#4904	Rabbit	Monoclonal	1:1,000
**SOCS3**	Abcam	ab16030	Rabbit	Polyclonal	1:500
**IL10**	Abcam	ab34843	Rabbit	Polyclonal	1:500
**GAPDH**	Abcam	ab9485	Rabbit	Polyclonal	1:2,000

Table S2. Primer sequences used for qRT-PCR

Gene	Forward Primer (5'- 3')	Reverse Primer (5'- 3')	Product size (bp)
C-MYC	CGTAGTTGTGCTGATGTGTGG	CTCGGATTCTCTGCTCTCCTC	272
HES1	TTGGAGGCTACGAGGTGGTA	GCCCCGTTGGGAATGAG	64
IL-10	AAGACCCAGACATCAAGGCG'	CACGGCCTTGCTCTTGTTTT'	116
IL-22	TGAATAACTAACCCCCTTTCCCTG	TGGCTTCCCATCTTCCTTTTG	87
KLF4	CGAAGCCACACAGGTGAGAA	TACGGTAGTGCCAGGTCAGTTC	94
LGR5	GGTGACAACAGCAGTATGGACG	GAAGGTGAACACAGCACTGAATGAA	140
MUC2	ACCCGCTCTATGTCACCTTC	GGGATCGCAGTGGTAGTTGT	131
RPS9	GATTACATCCTGGGCCTGAA	ATGAAGGACGGGATGTTCAC	161

S1

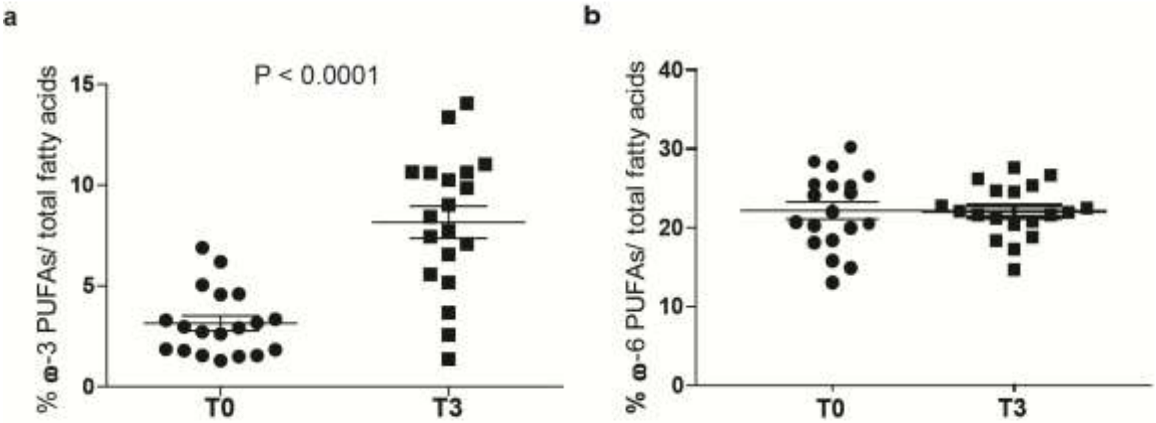

Supplementary Figure 1. (a) ω-3 PUFAs (EPA+DPA+DHA) and **b)** ω-6 PUFAs (arachidonic + linoleic acids) percentage in RBCs in all patients (n=19) at T0 and T3. Statistical significance was calculated using the paired two-tailed t-test. Data are shown as mean ± SEM.

S2

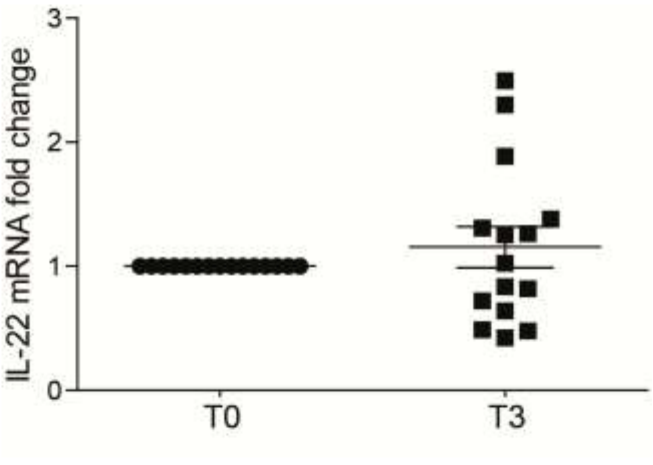

Supplementary Figure 2. mRNA expression levels of *IL-22* in compliant and responder patients (n =15) at T0 and T3. Data are shown as mean of square root transformed values ± SEM.

S3

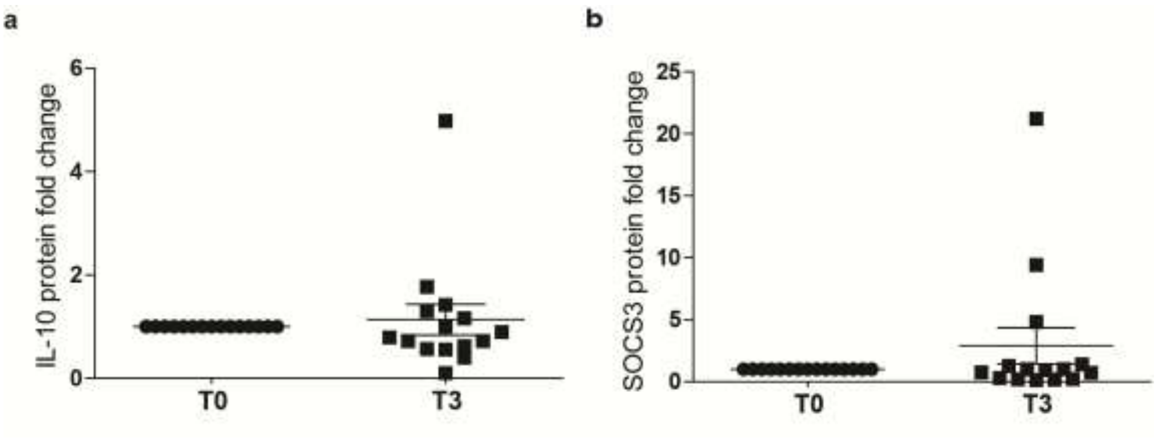

Supplementary figure 3. Protein levels of **(a)** IL-10 and **(b)** SOCS3 on homogenized sigmoid colon tissues in compliant and responder patients (n=15) at T0 and T3. Data are shown as mean of square root transformed values ± SEM.

S4

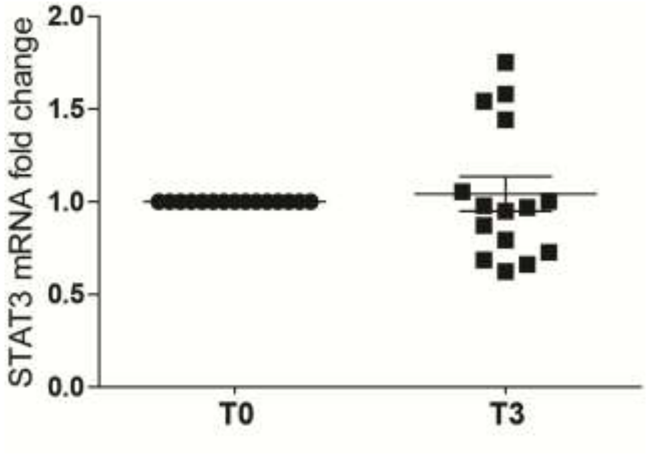

Supplementary Figure 4. mRNA expression levels of *STAT3* in compliant and responder patients (n =15) at T0 and T3. Data are shown as mean of square root transformed values ± SEM.

S5

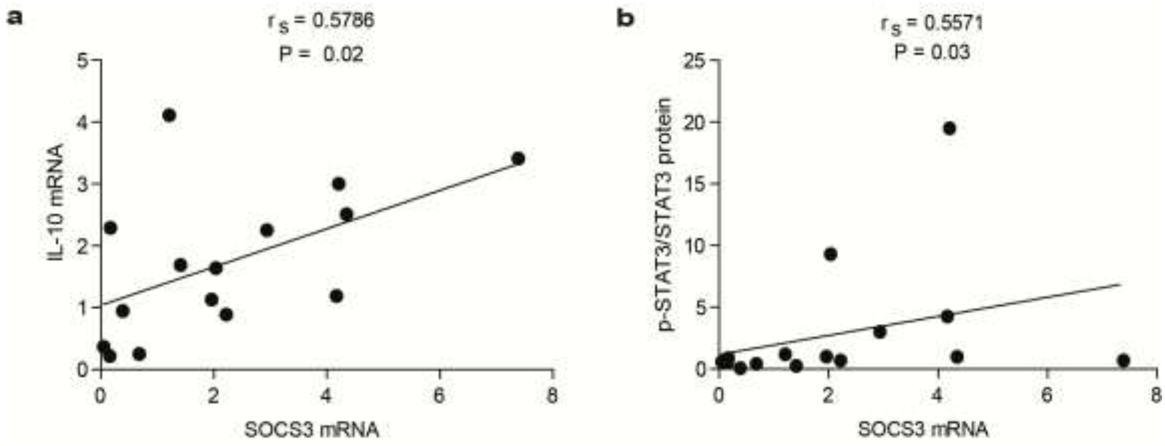

Supplementary Figure 5. Spearman's correlation analysis of *SOCS3* mRNA with **(a)** *IL-10* mRNA and **(b)** p-STAT3/STAT3 proteins in compliant and responder patients (n=15) at T3.

S6

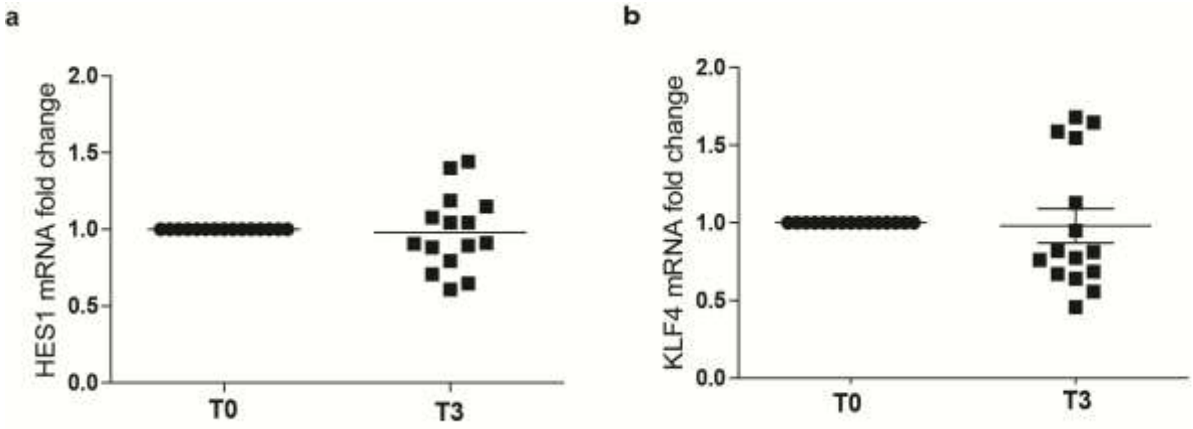

Supplementary Figure 6. mRNA expression levels of **(a)** HES1 and **(b)** KLF4 in compliant and responder patients (n =15) at T0 and T3. Data are shown as mean of square root transformed values ± SEM.

S7

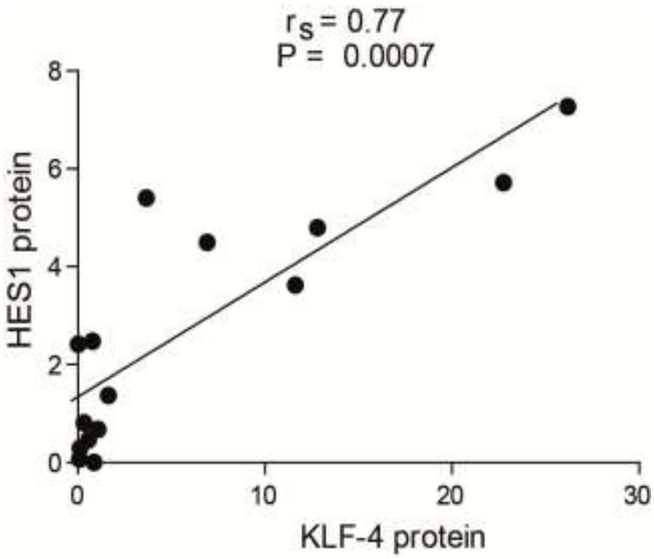

Supplementary Figure 7. Spearman's correlation analysis of HES1 and KLF-4 proteins in compliant and responder patients (n=15) at T3.

S8

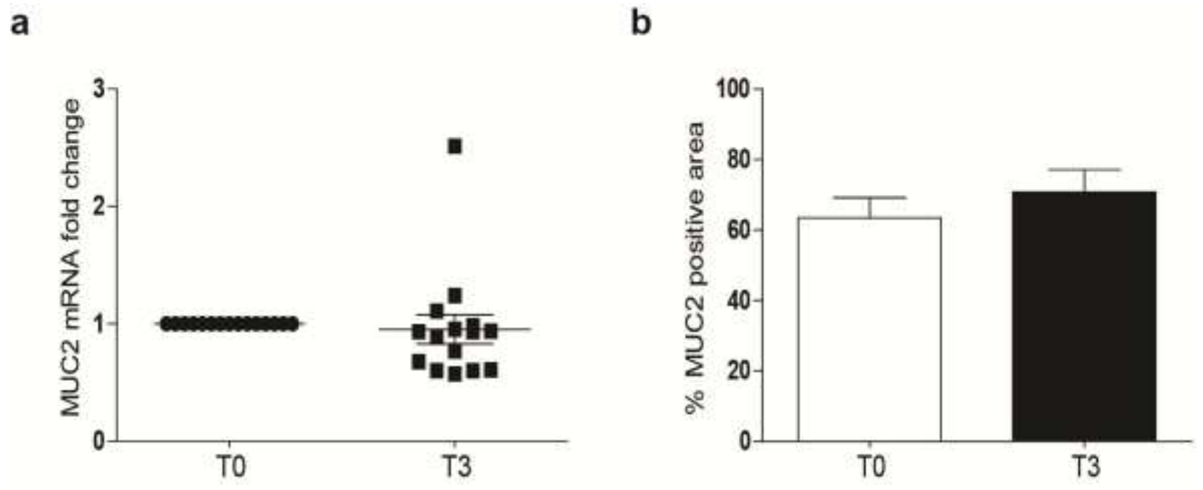

Supplementary Figure 8. (a) mRNA expression levels of MUC2 in compliant and responders patients (n =15). Data are shown as mean of square root transformed values ± SEM. (**b)** MUC2

protein analyzed by immunohistochemistry at T0 and T3 in compliant and responder patients (n =15). Data are shown as percentage of MUC2 positive area/total area.

S9

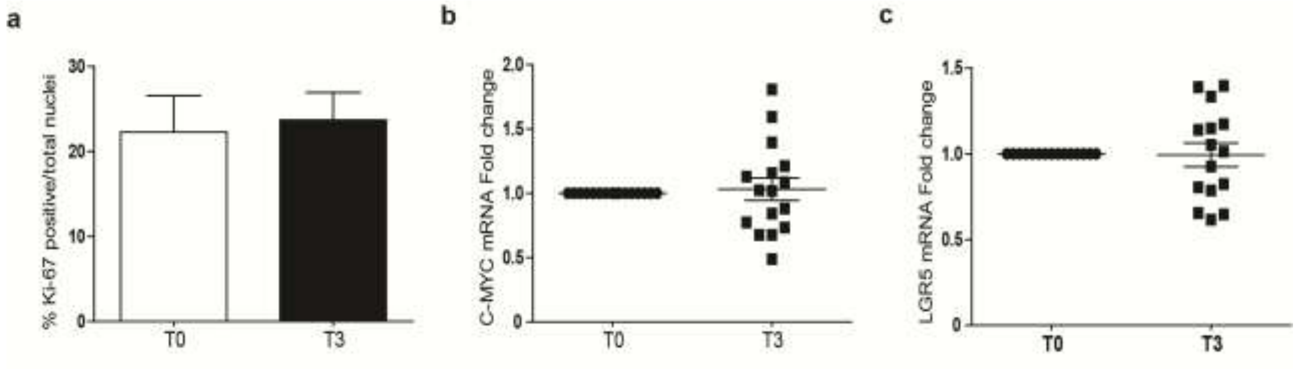

Supplementary Figure 9. (a) Ki-67 protein analyzed by immunohistochemistry at T0 and T3 in compliant and responders patients (n=15). Data are shown as percentage of Ki-67 positive/total nuclei. Expression of (**b**) *C-MYC* and (**c**) *LGR5* mRNAs in compliant and responder patients (n=15). Data are shown as mean of square root transformed values ± SEM.
